# The emergency department care of the cannabis and synthetic cannabinoid patient: a narrative review

**DOI:** 10.1186/s12245-021-00330-3

**Published:** 2021-02-10

**Authors:** Kevin M. Takakuwa, Raquel M. Schears

**Affiliations:** 1 Society of Cannabis Clinicians, PO Box 27574, San Francisco, CA 94127 USA; 2 University of Central Florida, Orlando, Florida USA

**Keywords:** Cannabis, Marijuana, Cannabinoid hyperemesis syndrome, Withdrawal, EVALI, Synthetic cannabinoids

## Abstract

**Background:**

Cannabis is the most prevalent illegal drug used and the second most common cause of ED drug-related complaints in the USA. Recently, newer more potent strains, concentrated THC products, and consumption methods have become available.

**Objective:**

Our first objective was to define cannabis use in the USA and provide a summary background on its current preparations, pharmacokinetics, vital sign and physical exam findings, adverse effects, and laboratory testing. Our second objective, using the aforementioned summary as relevant background information, was to present and summarize the care and treatment of the most commonly reported cannabis-related topics relevant to ED physicians.

**Methods:**

We first performed an extensive literature search of peer-reviewed publications using New PubMed and Cochrane Central Register of Controlled Trials to identify the most commonly reported cannabis-related topics in emergency care. Once the six topic areas were identified, we undertook an extensive narrative literature review for each section of this paper using New PubMed and Cochrane Central Register of Controlled Trials from the inception of the databases to September 30, 2020.

**Results:**

The six subject areas that were most frequently reported in the medical literature relevant to cannabis-related ED care were acute intoxication/overdose, pediatric exposure, cannabinoid hyperemesis syndrome, cannabis withdrawal, e-cigarette or vaping product use-associated lung injury (EVALI), and synthetic cannabinoids.

**Conclusion:**

As cannabis becomes more widely available with the adoption of state medical cannabis laws, ED-related cannabis visits will likely rise. While cannabis has historically been considered a relatively safe drug, increased legal and illegal access to newer formulations of higher potency products and consumption methods have altered the management and approach to ED patient care and forced physicians to become more vigilant about recognizing and treating some new cannabis-related life-threatening conditions.

## Introduction

Cannabis is the most widely used illegal substance in the USA [1] and worldwide [2]. There were an estimated 40.9 million current USA users in 2017 (15% of the population) and 192 million worldwide users in 2016. Lifetime usage in 2017 was estimated at 122,943,000, or 45.2% of the US population, a statistically significant increase from the previous year. There were 25,997,000 estimated to have used cannabis within the past month in 2017 or 9.6% of the US population, a statistically significant increase from the prior year.

The Drug Abuse Warning Network (DAWN) reported 455,668 ED visits related to cannabis in 2011 [3]. This equated to 146 ED visits per 100,000 population, second only to cocaine-related visits. If compared as a percentage of total ED visits (136,296,000 in 2011 [4]), then ED cannabis visits would be an estimated 0.3% of all ED visits. DAWN also noted that while synthetic cannabinoid reports appeared first in 2009, there were not enough cases to report. By 2011, 9.2 ED visits per 100,000 population were seen.

The National Hospital Care Survey estimated 29,230 ED visits for cannabinoids in the USA during 2013 using a sample of 82 of 581 hospitals [5]. Of these, an estimated 13,304 ED visits were more specifically related to substance use-related symptoms or procedures. Men were involved in 62% of cases compared to 37% involving women. The age range of 0–15 years old was 3–5% of all cases, 16–34 were 52–53%, 35–54 were 33%, and 55 and over were 10–11%. Over half 50–55% were discharged home, 35–44% were admitted or transferred, 2% left AMA, and 5–9% had some other disposition. In comparison to the other ten priority substances studied (alcohol under age 21, anti-depressants, antipsychotics, benzodiazepines/sedatives, cocaine, hallucinogens, heroin, opiates, CNS stimulants) in the USA, cannabis was the second most frequent ED-related substance behind opioids.

The 2018 National Poison Data System Annual Report cites 157 e-cigarette, 10,446 marijuana derivative (1082 concentrated extracts, 6040 dried plant marijuana, 1974 edible, 44 oral capsules or pills, 118 pharmaceutical preparations, 24 topical, 167 undried plants, 997 other/unknown preparations), and 1993 synthetic cannabinoid single substance pharmaceutical exposure cases out of 1,462,902 total pharmaceutical exposures [6]. This represents 0.7% for cannabis combined and 0.1% for synthetic cannabinoids. The report lists cannabinoids and analogs associated with 0.51% of single substance fatal exposures, 1.4% of total substances reported in fatal exposures and as the 22nd of 25 top fatality substance categories.

Cannabis use has existed for millennia and became legalized medically in California in 1996 after over half-a-century of prohibition [7]. Despite its continued illegal federal designation as a Schedule I drug since 1970 [8], 36 US states have legalized it for medical and 15 US states plus DC for adult use [9]. As more US states legislate legal medical as well as adult-use/recreational cannabis, ED physicians should expect more cannabis-related visits [10–13], particularly involving highly concentrated and unregulated products. In this article, we first provide a summary background on newer cannabis products, inhalation and ingestion pharmacokinetics, reported and expected vital sign and physical exam findings, adverse effects, and laboratory testing. This background facilitates understanding of six topic areas that we identify as most relevant to ED physicians.

## Methods

**Figure 1:**
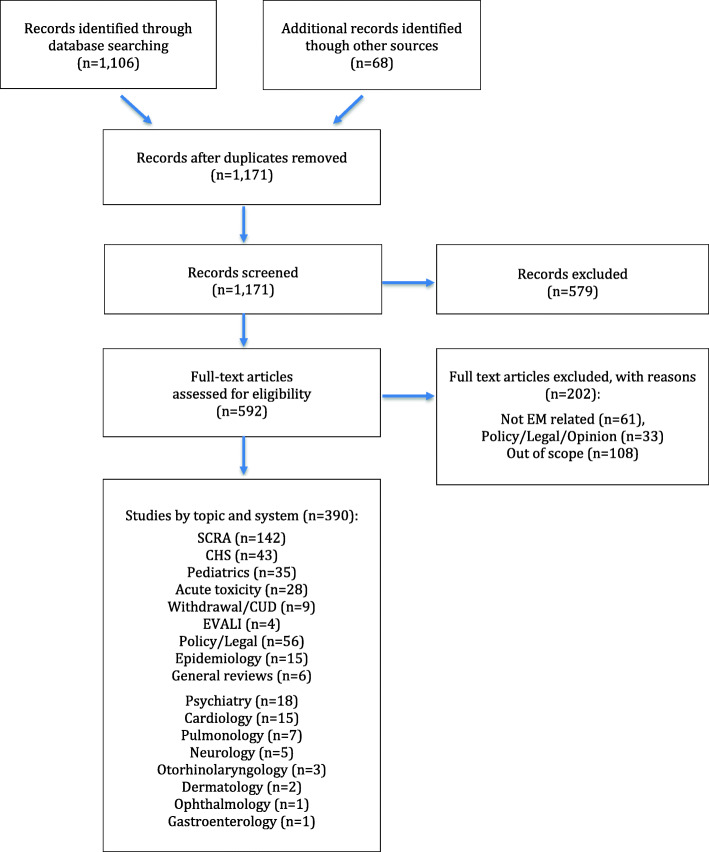
Flow diagram of how studies areas were selected

An extensive literature search of peer-reviewed publications was performed using New PubMed and Cochrane Central Register of Controlled Trials from the inception of the databases up to September 30, 2020. We started our search broadly in PubMed with the search term “cannabi*” or “marijuan*,” which resulted in 55,816 results. We eliminated 23,051 that were not in English or were not in humans, leaving 32,765. After adding the search term “emergen*,” we were left with 1106 articles. After adding in Cochrane searches, we employed a Preferred Reporting Items for Systematic Reviews and Meta-Analyses search strategy to identify the most commonly reported topic areas related to the ED care of patients exposed to cannabis (Fig. 1).

We found that synthetic cannabinoid-receptor agonists (SCRA), cannabinoid hyperemesis syndrome (CHS), pediatric cases, acute toxicity and overdose, cannabis withdrawal/cannabis use disorder (CUD), and e-cigarette, or vaping, product use-associated lung injury (EVALI) were the most commonly reported topic areas related to ED cannabis care in our initial search strategy. Because CUD may lead to withdrawal, and reports of ED care were more related to withdrawal, we combined the topic into one and simply labeled it as “withdrawal.” We also found that the effects of cannabis reported in papers focusing on specific organ systems were most reported for the psychiatric and cardiac systems.

For each of the 6 subject areas using the PubMed search for the most recent sort, we found 1782 articles for “(cannabi* or marijuan*) and synthetic,” 1345 articles for “(cannabi* or marijuan*) and pedia*,” 1063 articles for “(cannabi* or marijuan*) and intox*,” 895 articles for “(cannabi* or marijuan*) and withdrawal,” 170 articles for “(cannabi* or marijuan*) and hyperemesis,” and 48 articles on “EVALI.” Focusing on articles related to human clinical studies, we gave the highest preference for inclusion in this paper to recent meta-analysis papers, systematic review articles, evidence guidelines, randomized controlled trials, national database reports, journals with higher impact factors, articles related to ED care, and studies published on populations from the USA, respectively. We reviewed titles of papers and abstracts first before reading full articles to determine relevance and inclusion into each section of the paper. We additionally hand searched for relevant reports in references to all related articles. A consensus process between co-authors was used to discuss all relevant topics and published studies for inclusion or exclusion into this paper. Due to the varied protocols, quality of studies, publication biases, length of studies, difficulty in studying and quantifying cannabis use in light of its federal scheduling, and paucity of studies, we did not believe that any other study design including meta-analysis, systematic review, or evidence guidelines or grading of available literature was possible for this subject matter. We therefore determined that a narrative review was most appropriate for this important topic area that would include existing available literature across medical specialties.

### Summary background

#### Cannabis preparations

Cannabis’ most notorious constituent, delta-9-tetrahydrocannabinol (THC) that gives cannabis its psychoactive properties, was identified in 1964 [14]. Recent decades have seen remarkable increases in the average THC content percentage of cannabis (from 3% in the 1980s to 4% in 1995 to about 12% in 2012–2014) as well as hashish and hash oil [15, 16] probably due to selective breeding. Newly developed consumption formats now exist besides the traditional botanical usage (i.e., smoking the flower, e.g., in joints, hand or water pipes, vaporizers) that broadly includes edibles (e.g., gummies, chocolates, baked goods, beverages) and extracts (aka concentrates). Methods for making concentrated cannabis, historically done by compressing cannabis flower resins into hash or hashish, have advanced. It can now be produced by utilizing solvents such as alcohol, ethanol, butane, propane, or CO_2_ to extract and concentrate THC into newer formats including tinctures, oils, shatter, wax, and budder, which can be eaten (e.g., directly, combined with other substances, or placed in capsules), inhaled (e.g., smoked or vaporized through dabbing or vaporization pens), or used topically or rectally. As such, cannabis is now more broadly available in a variety of formats and concentrations making consumption easier and less conspicuous.

#### Inhalation and ingestion pharmacokinetics

The most common modes of cannabis usage are inhalation and ingestion. The psychotropic effects of inhaled THC usually begin in seconds to minutes, plateau in 15–30 min, and subside within 2–3 h. With ingestion, the psychotropic effects typically begin in 30–90 min, plateau after 2–3 h, and remain for 4–12 h [17]. While these time frames are variable, dose-dependent, and were described before the newer concentrated cannabis products were developed, it gives the ED physician a general sense of the expected duration of effects over time.

#### Vital sign and physical exam findings

Reported changes in vital signs and physical exam findings that can occur in adults as a result of cannabis use are noted below. While this is a brief summary list of common changes, we note these so that clinicians may be aware of potential effects. However, there are no absolute changes that rule in or rule out cannabis use so any vital sign or physical exam abnormality should be thoughtfully considered (Table 1).

**Table 1:** Summary list of possible vital sign and physical exam findings seen in adults after cannabis use

Heart rate: cannabis can cause tachycardia, possibly due to increasing the sympathetic nervous system, vagal inhibition or vasodilation causing reflex tachycardia [17–23].^a^	
Blood pressure: cannabis can cause hypertension but may lead to orthostatic hypotension by causing peripheral vasodilation [17, 18, 20, 24].	
Respiratory rate: cannabis may cause tachypnea [25].^b^	
Temperature: cannabis may cause hypothermia [17, 18].	
Eyes: conjunctival injection, diminished tears. There are case reports of blurry vision and nystagmus [17, 26, 27]^c^	
Mouth: dry mouth [17]	
Respiratory: bronchodilation [17, 22, 27, 28]^d^	
Neurologic: slurred speech, disattention, decreased concentration, motor delay, ataxia, weakness, heightened sensation, drowsiness [17, 27, 29, 30]^e^	
Psychiatric^%^: euphoria/dysphoria, anxiety/anxiolysis, confusion, hallucinations, exacerbation of psychotic states, sleep [17, 30]	

^a^For pediatric patients, tachycardia has more commonly been reported but bradycardia has additionally been observed [31]

^b^For pediatric patients, hypoventilation may be seen [31]

^c^For pediatric patients, dilated pupils are commonly reported but midpoint and pinpoint are also reported [31]

^d^For pediatric patients, respiratory depression and hypoventilation have been reported [31]

^e^For pediatric patients, seizures, lethargy, hypotonia, and altered mental status have been reported [31]

% The psychoactive properties of cannabis were the main focus of early research [32]

In an observational study of acute cannabis exposures reported to two poison control centers, tachycardia was seen 55% (77/140), bradycardia 5% (7/140), hypertension 33% (27/82), hypotension in 9% (7/82), and hypoxemia 5% (3/55) across all age groups. Tachycardia was more commonly observed in both adolescents and adults (46%) when cannabis was inhaled compared to other exposures (RR 1.58) [33] and in those who ingested edibles compared to concentrates [34]. Additionally, CNS excitation totaled 38% (that included anxiety/paranoia/panic attack (22%), agitation/irritability/aggression (9%), hallucination (5%), seizure (4%), tremor/myoclonus (3%) and psychosis (2%)), CNS depression totaled 25% (that included reduced consciousness (23%), weakness/impaired coordination (7%), syncope/fall/found down (4%), obtunded/comatose/unresponsive (2%), speech abnormality (2%) and confusion (2%)), and other neurotoxicity totaled 33% (that included lightheadedness/dizziness/vertigo (14%), altered (10%), dysphoria (4%), unusual/unexpected sensation (4%), numbness/tingling (2%), euphoria (1%), and headache (1%)) across all age groups [33]. Neuroexcitation was more commonly reported for inhalation compared to ingestions (RR 1.54).

#### Adverse effects of medical cannabis

A systematic review of the safety of medical cannabinoids covering over 40 years was performed [35]. Thirty-one studies were identified that used medical cannabis: 23 randomized controlled trials (including 2698 patients) and eight observational studies. A total of 4779 adverse events were reported: 164 serious adverse events and 4615 non-serious adverse events. There was no evidence that medical cannabinoids caused any serious adverse events (fatal or non-fatal) compared to controls. There were, however, significantly more non-serious adverse events in the cannabinoid compared to the control group. We report this data because it represents common complaints seen with the use of medical cannabis (oral and oromucosal preparations) but note that this study did not include smoked, inhaled, or other newer forms of cannabis concentrates so the effects are not generalizable to all forms of currently available cannabis (Table 2).

**Table 2:** Percentage of non-serious adverse events for medical cannabinoids (oral and oromucosal preparations) compared to control group

System/organ class	Percentage of non-serious adverse events	
	cannabinoid group	control group
Nervous system disorders^a^	36.7	31.1
Gastrointestinal disorders^a^	16.4	15.0
General disorders	14.1	17.9
Psychiatric disorders^a^	11.1	7.4
Musculoskeletal disorders	7.2	10.6
Renal and urinary disorders	5.1	8.2
Infections	2.9	4.3
Eye disorders^a^	2.3	1.0

^a^Significant difference

The remaining systems organ classes including: injury, poisoning and procedural complications; metabolism and nutritional disorders; respiratory, thoracic and mediastinal disorders; skin and subcutaneous tissue disorders; blood and lymphatic system disorders; cardiac disorders; vascular disorders; and investigations all reported < 1% non-serious adverse events in the cannabinoid group and were not significantly different from the control group.

Under each subcategory for specific disorders the following complaints without respect to the control group were made:

For nervous system disorders, the most common complaints in the cannabinoid group were dizziness (15.5%), somnolence (8.2%), sedation (2.2%), headache (1.7%), abnormal coordination (1.6%), tremor (1.5%) and paresthesias (1.4%).

For gastrointestinal disorders, the most common complaints in the cannabinoid group were other gastrointestinal tract disorder (6.2%), dry mouth (5.2%) and nausea (2.5%)

For general disorders, the most common complaints in the cannabinoid group were pain (6.0%), asthenia (4.3%) and fatigue (2.4%)

For psychiatric disorders, the most common complaints in the cannabinoid group were euphoric mood (2.8%), depression (2.0%) and mental disorder (1.1%)

For musculoskeletal, connective tissue and bone disorders, the most common complaint in the cannabinoid group was muscle spasm (6.3%)

For renal and urinary disorders, the most common complaint in the cannabinoid group was bladder disorder (4.8%)

For infections and infestations, the most common complaint in the cannabinoid group was infection (2.9%).

For eye disorders, the most common complaint in the cannabinoid group was blurred vision (2.3%).

All remaining subcategories of complaints were reported at < 1%

Table adapted from Wang T, Collet J-P, Shapiro S, Ware MA. Adverse effects of medical cannabinoids: a systematic review. CMAJ. 2008; 178(13): 1669–1678

Recently, increasing studies [36] question the half-century reported association between using cannabis and psychosis and schizophrenia [37, 38]: specifically, does cannabis use cause psychosis or are schizophrenics predisposed to using it [38–41]. One systematic review and meta-analysis of seven studies between 1966 and 2004 concluded that cannabis is an independent risk factor for psychosis and psychotic symptoms [42]. Another meta-analysis of ten studies totaling 66,816 individuals that examined the relationship between cannabis use and risk of psychosis found further support for high cannabis levels increasing the chance of psychotic outcomes and the risk of psychosis associated with higher levels of use [43]. A multicenter case-control study of 901 European patients with first-time psychosis found three-fold increased odds of psychosis in daily cannabis users compared to never users that increased to almost five-fold when using high-potency cannabis [44]. They concluded that the frequency and type of cannabis contributed to the highly variable incidence of psychosis among their study sites.

In contrast, a critical review of the relationship between cannabis and psychosis reports no evidence that cannabis causes psychosis [36]. Instead, the authors conclude that individuals vulnerable to psychosis are more likely to use cannabis at a younger age and use more heavily than those not vulnerable to psychosis. Thus, while there appears to be no conclusive evidence on whether cannabis causes psychosis, ED physicians should consider the possible relationship in an undifferentiated psychotic patient, particularly if that patient is a daily or high-potency cannabis user, until further evidence is elucidated.

#### Laboratory testing for cannabis

The most commonly used ED method to screen for cannabis is a urine drug screen (UDS), which uses an immunoassay to detect the delta-9-THC metabolite 11-nor-delta-9-tetrahydrocannabinol-9-carboxylic acid (9-carboxy-THC). 9-carboxy-THC can be detectable within minutes in the plasma after smoking THC but can take several hours when ingested. It is typically detectable for 1–2 days up to 4 days in infrequent cannabis users but may last 2 weeks in daily users and over 4 weeks in long-term heavy smokers [45]. In contrast, 9-carboxy-THC can be detectable within 1 h in the urine after consuming THC. A positive urine test is non-specific for the time of use and can indicate cannabis use from one hour up to several weeks [46–49]. Some substances have been reported to cause false-positive urine tests including hemp containing foods, proton pump inhibitors, promethazine, dronabinol, some NSAIDs, and efavirenz [45, 50]. THC can be confirmed by gas chromatography, mass spectrometry, or high-performance liquid chromatography. It can additionally be detected in the blood, saliva, or hair testing, but these are neither rapid nor appropriate for ED usage.

## Results

### ED cannabis presentations

#### Acute intoxication/overdose

##### Clinical scenario

A euphoric woman presents to the ED with concerns about her 4-year-old son who has been sleepy and not waking up at home. You notice the smell of cannabis on her clothing as she begins to tear up while divulging that some of her hash brownies are missing.

A study of 253 acute exposures to cannabis (without co-ingestants) were reported to the Oregon/Alaska Poison Center over 16 months [33]. The median age was 20 years (range 8 months–96 years), males 54%, and children < 12 years were 28%, adolescents 12–17 years were 17%, and adults > 18 years were 55%. Children compared to adults were more likely to unintentionally (99% vs. 11%), ingest (97% vs. 69%), homemade (35%) vs. retail (40%), edibles (65% vs. 49%), and experience sedation (52%) vs. neuroexcitation (47%). Children obtained cannabis from a family member/caretaker 73%. Adolescents had characteristics of both children and adults.

Adults compared to children were significantly more likely to experience CNS excitation (47% vs. 18%, which included anxiety/paranoia/panic attack 33% vs. 1%), other neurotoxicities (41% vs. 18%, which included lightheadedness/dizziness/vertigo 19% vs. 3% and dysphoria 6% vs. 0%), cardiac symptoms (22% vs. 1%, which included palpitations 18% vs. 0%), and gastrointestinal symptoms (31% vs. 10%, which included nausea/vomiting 13% vs. 0% and nausea without vomiting 7% vs. 0%). Adults were more likely to stay at home (28% vs. 14%). There was one reported adult requiring intubation and one death.

Children compared to adults were significantly more likely to experience CNS depression (52% vs. 24%, which included reduced consciousness 45% vs. 8% and obtunded/comatose/unresponsive 6% vs. 1%) and be explicitly asymptomatic (16% vs. 2%). Children were more likely to be admitted to the ward (20% vs. 4%). There was no difference in ED visits with discharge to home or ICU admission. There were two reported intubations (in a 9 month and 1 year old) and no reported deaths in children. The authors report that concentrated products were significantly associated with intubation, particularly when ingested by children.

In a retrospective chart review of a Colorado ED, 238 of 2567 visits at least partially attributable to cannabis were related to cannabis edibles [51]. Edibles as compared with inhaled cannabis were more associated with intoxication (48% vs. 28%), acute psychiatric symptoms (18% vs. 11%) and cardiovascular symptoms (8% vs. 3%). Inhaled compared to edible cannabis was more likely to be associated with CHS (18% vs. 8%).

In a case study involving 12 children and 9 adults (age range 6 to 60 years) who inadvertently ingested THC gummies at a child’s birthday 1 h prior to ED arrival [52], of the 12 pediatric patients: 83% had tachycardia; 66% had hypertension; 58% had visual changes; 50% were dizzy or lethargic; 42% were confused, had nausea/vomiting or tachypnea; 25% had dry mucous membranes; 17% had abdominal pain, anxiety, conjunctival injection, or hallucinations; and 8% had headache, increased tone, lightheadedness, palpitations, paranoia, or dyspnea. Laboratory testing revealed 58% had elevated lactate and 42% had leukocytosis. Of the 9 adult patients: 67% had dizziness; 44% had nausea/vomiting or palpitations; 33% had dry mucous membranes, tachycardia or visual changes; 22% had anxiety, confusion, conjunctival injection, or dyspnea; and 11% had abdominal pain, flushing, headache, hypertension, lethargy, or sore throat. Laboratory testing showed 11% had elevated lactate or leukocytosis. While over half of patients had a UDS done, 100% tested positive. For 21 who had serum THC levels taken, 16 were positive though not available to ED staff since this was a send-out test. For the 12 pediatric patients: 5 were admitted and 7 discharged home. Of the 5 admitted, all were discharged home within 12 h. In contrast, none of the adults were admitted.

Cannabis intoxication has been defined by the Diagnostic and Statistical Manual of Mental Disorders (DSM-5) [53] (Table 3). These aforementioned studies demonstrate that children and adults respond very differently to acute cannabis intoxication, which appears to be influenced by the mode (i.e., inhalation vs. ingestion) and type of consumption (botanical vs. concentrated products) due to the pharmacokinetics of absorption and likely the relative amount of THC per kilogram of body weight. While there is a good likelihood that abnormal vital signs and physical exam findings are observed, there are no pathognomonic findings in acute cannabis toxicity. UDS provided a clue in the undifferentiated pediatric patient with symptoms of cannabis intoxication. Other laboratory testing did not appear helpful in simple cannabis ingestion in healthy people without concern for co-ingestants.

**Table 3:** Cannabis intoxication criteria^a^

The DSM-V defines cannabis intoxication as:	
1. Recent cannabis usage	
2. Significant behavioral or psychological change that occurs during or immediately after cannabis use	
3. Two or more of the following findings occurring within 2 hours of usage and not attributable to another cause: tachycardia, conjunctival injection, dry mouth, increased appetite	
Note: Intoxication can occur with or without perceptual disturbances	

^a^Table adapted from American Psychiatric Association: Diagnostic and Statistical Manual of Mental Disorders: Diagnostic and Statistical Manual of Mental Disorders, Fifth Edition. Arlington, VA: American Psychiatric Association, 2013

The general treatment for acute cannabis intoxication in adults is supportive care. Consider placing and monitoring the patient in a quiet area of the ED. If the patient is having mild psychiatric symptoms but not demonstrating harmful or self-injurious behavior, the clinician should consider a one-to-one observation. If moderate to severe psychiatric symptoms evolve during the ED visit, then maintaining patient and care-provider safety is paramount and involving psychiatric consultation is warranted. The effects of acute intoxication in adults tend to be self-limiting and typically do not require hospitalization, with exceptions for acute psychosis and medical conditions that may be triggered or precipitated by cannabis use. We recommend early discharge when the adult patient can ambulate, has normal cognition and judgment, and can be sent home with a reliable caretaker.

The ED physicians should always consider the age group, mode of consumption, and type of product used in evaluating acute intoxication, keeping in mind that some patients (particularly infants, see below) may require emergent intubation. Utilizing the state poison control center is encouraged, especially for any unexpected abnormalities as well as concerns for unique cannabis preparations and co-ingestants.

##### Clinical scenario

A 30-year-old previously healthy man is brought into the ED from the airport after he could not be woken up to disembark from his return flight from Amsterdam. His companion reports he attended an international music festival prior to boarding the plane.

A study of the European Drug Emergencies Network (Euro-DEN) project collects ED data from patients with acute toxicity from using recreational drugs [54]. During their 6 months of data collection (October 2013–March 2014), there were 356 toxic episodes out of 2189 that involved cannabis. Of 356 toxic episodes reported, the majority were male (76%), median age 26, and residents of the research center city (77%). The majority, 320 (90%) used other substances: 59% alcohol, 23% benzodiazepines, 18% cocaine, 11% amphetamines, and 7% new psychoactive substances. Only 36 (10%) involved an exposure to cannabis alone. Of those, 71% received no treatment and 29% received sedation, primarily benzodiazepines. The majority (77%) were discharged, 9% left the ED, and 14% were admitted (4 of the 5 to psychiatry). They report one death in an 18-year-old male with seizure disorder non-compliant with his anti-convulsants who collapsed while smoking cannabis at a bar. His death was attributed to a cardiac rhythm disturbance. They conclude that the majority of patients had self-limiting mild neuropsychiatric symptoms and/or vomiting that required no treatment and a short ED stay.

A study of three Swiss hospitals found that 26% of 717 ED visits for cannabis were due to cannabis use alone [55]. These 186 patients presented with complaints of nausea/vomiting 26%, palpitations 25%, anxiety 23% and chest pain 15%. The majority (83%) were discharged home. Severe complications included psychosis 7%, coma 6%, seizures 5% and one patient required intubation. The 531 co-ingestant patients were more likely to have impaired consciousness, agitation, respiratory depression and hallucinations and be admitted to intensive or psychiatric care.

Another study used the electronic Canadian Hospitals Injury Reporting and Prevention Program (eCHIRPP) database, which is a web-based injury and poisoning surveillance system in 11 pediatric and six general EDs in Canada [56]. They abstracted data from 2011 to 2016 and found 184 cases per 100,000 eCHIRPP cases involved cannabis use. The commonest related injury was unintended (e.g., motor vehicle collision, poisoning, and hallucination, 66.8%) followed by physical assault/aggression (e.g., altercations, 13.0%) and self-harm (e.g., suicidal thoughts, suicidal attempts, depression, 12.1%). The authors report that the external causes of the above injuries were associated most commonly with intoxication (69.4%), poisoning (68.5%), falls (9.7%), and assault (9.4%). Cannabis was used alone in 72.4/100,000 cases and with alcohol in 74.6/100,000 cases, illicit drugs in 11.3/100,000 cases, and medications in 7.9/100,000 cases. Cannabis used alone was not associated with hospitalization, but it was when used with medications and/or drugs. While the authors conclude that cannabis use injuries are rare and most commonly unintentional, they highlight some of the concerns with cannabis use and potential issues faced by ED physicians.

The importance for the clinician is to recognize that the patterns of cannabis use vary around the world and by region. The typical profile of a patient—a young male—predominates throughout the world. The majority of patients in Europe were safely discharged to home, particularly if cannabis was the only substance used. A clinician must always consider co-ingestants including alcohol, medications, and other drugs or substances (e.g., opioids, benzodiazepines, stimulants) often used in combination when evaluating the ED patient, be aware of potential associated physical injuries and always assess for self-harm or psychiatric states as a cause or result of usage.

##### Clinical scenario

An anxious 34-year-old man presents to the ED complaining of sharp diffuse chest pain that started 30 min after smoking a joint from an acquaintance. He feels tingly all over, short of breath, and the same panic he had the last time he tried cannabis. He is feeling no better now but wants to go home.

There are a number of case reports associating cannabis use with possible cardiac symptoms or events such as chest pain, arrhythmias, acute myocardial infarction (MI), and death [57–60]. A recent systematic review attempting to determine cardiovascular risk with any cannabis use including Sativex, Dronabinol, and Nabilone (cannabis-derived, synthetic cannabis, and synthetic cannabis-like medications) found 116 subjects from mostly case reports and observational studies [61]. Of 116 who had used cannabis, the authors documented 33 MIs, 27 ischemic strokes, 5 cases of thromboangiitis obliterans, 4 hemorrhagic strokes, 2 myopericarditis cases, and 11 deaths from cardiovascular disease. While the investigators concede that there is no unique mechanism to suggest a relationship between cannabis and cardiovascular disease, they nonetheless concluded that a relationship exists between cannabis and cardiovascular disease, more so for ischemic strokes, and that there may be a link between cannabis and cardiovascular consequences especially with higher doses.

The Determinants of Myocardial Infarction Onset Study interviewed 3882 patients with acute MI an average of 4 days after MI [62]. They concluded that the risk of MI onset was increased 4.8× over baseline in the 60 min after cannabis use but the elevated risk rapidly decreased thereafter. However, an 18-year follow-up of this same study cohort found a non-significant trend between cannabis users and increased mortality [63]. A recent review covering 33 studies reported that 28 of the studies found an increased risk of an acute coronary syndrome (ACS) with cannabis usage [64] but noted that just 5 of the 33 were designated level I systematic reviews: 3 supported while 2 refuted [65, 66] the association (and none of the reports studied the direct relationship between cannabis and ACS). One of the latter studies was a comprehensive review to determine the relationship of cannabis use to cardiovascular risk factors and clinical outcomes [65]. They located 13 studies on cardiovascular risk factors that included diabetes, obesity, and dyslipidemia and 11 studies on clinical outcomes including acute MI, cardiovascular mortality, and stroke. These authors found insufficient evidence of a relationship between cannabis and cardiovascular risk factors and clinical outcomes citing various methodological limitations in their cited study designs.

Whether a mechanism of injury exists between cannabis use and ACS is unknown though multiple theories exist [67–69]. While there are many reports that implicate THC as a cause of cardiac deaths, we would hesitate to make any firm conclusions. Recently, a 70-year-old man with known coronary artery disease presented to an ED after he ate a THC lollipop [70]. This was his first experience with cannabis, and the dose was estimated to be 70 mg, which is 7 times the single serving size of an edible allowed by California [71] or recommended by Colorado [72]. After his ingestion, he developed the fear of dying and chest pain 30 min later. He was ultimately diagnosed with a non-ST-elevation MI, and his chest pain subsided after his hallucinations resolved. The authors’ surmised that the hallucinations were sympathomimetic and precipitated his MI.

This case as well as other evidence [20, 67, 73, 74] illustrates the possibility that while cannabis may not have directly caused cardiovascular events, the resulting hemodynamic effects of cannabis use that transiently raise heart rate and blood pressure may have precipitated them in patients with existing structural heart disease. Furthermore, there are case reports involving cannabis consumption associated with acute MI in those with normal coronaries and of young men, usually with underlying structural heart disease that can be brought on by exercise [61]. Another possibility is that cannabis usage may be associated with a higher risk of cardiac dysrhythmias, though rare could potentially be life threatening, as was reported in a recent systematic review [75].

Therefore, if a patient using cannabis presents with any cardiac symptoms, particularly one with known structural heart disease, we would recommend treating the patient as a potential cardiac patient first and foremost keeping in mind that even young people may have undiagnosed structural heart disease or could have a dysrhythmia. We propose performing a sound cardiac workup that considers an ECG, cardiac markers and a chest X-ray rather than attributing or disregarding cannabis as the culprit of their symptoms without the need for further workup, recalling that palpitations and chest pain are common complaints with cannabis use [33, 51, 55]. Future research may reveal prognostic cardiovascular responses in children and adults when using varying modes, quantities and types of cannabis.

#### Pediatric exposure

##### Clinical scenario

A lethargic infant is brought to the ED by concerned parents. They report that she has not woken up or eaten since they returned home from visiting relatives. She was fine when they dropped her off at the babysitter this morning but now looks “off.” She is unable to sit up on her own and her O_2_ saturation is 95% on room air.

A systematic review of unintentional cannabis ingestion in children aged < 12 years old located 44 articles that included 3582 children [31]. Ten studies documented lethargy as the most common complaint. In 44 case series and reports that included 114 children, lethargy was reported in 71% and ataxia was observed in 14%. The authors report 38% ingested cannabis resin, 13% ingested cookies, and 13% smoked cannabis joints. Patients were commonly observed to have tachycardia, mydriasis, and hypotonia. The mean length of stay was 27.1 + 27 h, 18% were admitted to a PICU, and 6% were intubated.

While there is no evidence of any mechanism of action that links cannabis with respiratory depression, we would surmise that the over sedating effects from high doses of THC could cause loss of muscle tone, mechanical airway narrowing, or even upper airway obstruction in the relatively narrower pediatric airway. We suspect this since 6 of the 7 patients were aged 2 and under (8, 9, 10, 15, and 19 months old and 2 years old) while the seventh patient was 3 years old.

A study in France reported that of 235 children admitted for cannabis intoxication through 24 pediatric EDs over 11 years, 72% ingested hashish and 8 required assisted ventilation [76], supporting the idea that high THC concentrations may be a risk factor for hospitalization and for airway compromise. While we believe that intubation has not been common historically for cannabis exposed pediatric patients, this may be changing. With the combination of increasing availability and access to cannabis products through state legalization [77], higher THC concentrations of cannabis botanicals being grown [16], and the introduction of and familiarity with cannabis concentrates [78], more accidental toxic exposures to pediatric patients seems inevitable [33, 79–82]. We therefore would advise clinicians to immediately assess for CNS depression and any signs of respiratory compromise in a pediatric patient, particularly in infants and young toddlers exposed to concentrates, and maintain a low threshold for intubation to avoid respiratory insufficiency as a potential complication.

In a retrospective review study comparing first-time cannabis users with cannabis experienced children aged 1 month to 20 years old who were treated for acute cannabis toxicity, the authors found that patients who had never used cannabis before were more likely to feel lethargic or somnolent, have longer-lasting symptoms, and required longer hospital stays [83]. They report patients were more likely to need hospital admission with > 5 mg THC/kg and were more likely to be observed and discharged from the ED with 3 mg THC/kg or less. While there were significant study limitations (small sample size, the two groups were very different in age (3.5 vs. 15.1 years. old) so had significant weight differences and they were unable to accurately estimate the THC dose between groups (26% vs. 5%)), the study does provide some guidance on what to expect if the clinician knows the amount of THC consumed in a pediatric patient. As a reference, another paper on administration and dosing guidelines for medical cannabis recommends that the total daily dose of THC should be limited to 30 mg/day or less, preferably combined with CBD [84].

We hope that trends of pediatric exposures to cannabis will diminish over time as state laws strengthen to protect children from unintended exposures. For example, now some state cannabis regulations are requiring child-resistant packaging, imposing limits on the total amount of THC per package and quantity of THC per serving size, and banning the candy-like appearance of edibles [85, 86].

For pediatric patients, the clinician may not have a reliable historian due to the age of the patient and the effect of THC on the executive functioning of the individual. The clinician should take a more detailed history from parents, guardians, or friends of non-obvious or suspected ingestions, for example, gummies, brownies, cookies, and candy, particularly in states that do not require child-resistant packaging. Asking parents or guardians if they have a medical cannabis card may provide hints. There does not seem to be a reliable sign or symptom including the physical exam and vital signs that suggests cannabis intoxication so a high level of suspicion should be raised for an undifferentiated patient with an acute onset of symptoms suggestive of CNS depression such as reduced consciousness, obtundation, unresponsive, or comatose [87]. A quick positive urine drug screen may hint at an early leading diagnosis in the appropriate context without other obvious considerations. We would still nonetheless encourage as part of the standard altered mental status workup a glucose, electrolyte panel, alcohol level, UDS, and consider a serum THC level and head CT, particularly if no improvement or any worsening occurs within 6 h. Clinicians should assess for cannabis extract ingestions, which often have a higher concentration of THC and therefore may cause more severe symptoms and be a risk factor for necessitating airway protection with intubation, which should be a low threshold consideration for infants and toddlers. Finally, the clinician should be aware that first-time ingestions and higher exposures of THC (> 5 mg/kg ingestions) might cause greater concern and relatively more symptoms of acute intoxication.

#### Cannabinoid hyperemesis syndrome (CHS)

##### Clinical scenario

A healthy-appearing 28-year-old man presents to the ED with his girlfriend reporting nausea, vomiting, and abdominal pain for the past 10 h. He vomited 15–20× overnight and “nothing is coming up anymore.” He has diffuse tenderness everywhere you touch though his abdomen is soft. His girlfriend confides, “We’ve been to 3 other emergency departments in the past 4 months and nobody can figure out what’s wrong.”

The earliest account of CHS comes from an article in 2004 reporting 19 South Australian patients with chronic cannabis abuse and cyclic vomiting [88]. For the nine cases detailed in the article, chronic cannabis use (“several years”) started before cyclic vomiting in all cases. Seven of nine patients who stopped cannabis had resolution of cyclic vomiting. Three patients who stopped cannabis and restarted it had a return of cyclic vomiting. Compulsive hot water bathing was reported in nine of ten patients.

A systematic review of published articles from January 2000 through September 2015 identified 2178 articles, reviewed 1253 abstracts and included 170 articles [89]. All were observational studies identified as low-grade quality studies. They summarize the diagnostic characteristics that may help ED physicians differentiate CHS from other nausea/vomiting/abdominal pain patients (Table 4). Their study reported abstinence from cannabis as the only definitive treatment in 96.8% (62/64). Among the 21 patients who continued cannabis use, 100% continued to have CHS symptoms.

**Table 4:** The diagnostic characteristics of cannabinoid hyperemesis syndrome^a^

Diagnostic characteristic	Frequency (%)
Severe nausea and vomiting	100
Cyclic vomiting over months	100
Age < 50 at onset of illness	100
Cannabis use at least weekly	97.4
Symptom resolution after stopping cannabis	96.8
Compulsive hot bathing that relieves symptoms	92.3
Abdominal pain	85.1
Reported daily cannabis use	76.6
Reported regular cannabis use > 1 year	74.8
Male predominance	72.9

^a^Table adapted from Sorensen CJ, DeSanto K, Borgelt L, Phillips KT, Monte AA. Cannabinoid Hyperemesis Syndrome: diagnosis, pathophysiology, and treatment-a systematic review. J Med Toxicol. 2017; 13(1): 71-87

CHS has since been formally defined by the Rome Foundation, a non-profit that supports the diagnosis and treatment of functional gastrointestinal disorders [90, 91] (Table 5). There are classically three phases of CHS: prodromal, hyperemetic, and recovery phase. The hyperemetic phase typically lasts 1–2 days [88, 92] and is when patients will likely present to the ED.

**Table 5:** Definition (Rome IV Criteria) of cannabinoid hyperemesis syndrome^a^

Starts after excessive and lengthy usage of cannabis for at least 6 months prior to diagnosis^b^	
- Must have recurrent vomiting (similar onset, duration and frequency) that appears like cyclic vomiting syndrome for at least 3 months	
- Resolution of vomiting after cannabis use stopped	
- All criteria must be met for the last 3 months	

^a^Table adapted from Stanghellini V, Chan FK, Hasler WL, et al. Gastroduodenal disorders. Gastroenterology. 2016;150:1380–1392

^b^Might have pathological related bathing behavior

To uncover the impact of CHS on EDs, data were taken from the National Emergency Department Sample records from 2006 to 2013 [93]. The authors searched out vomiting as the primary ICD-9 diagnosis with associated coding for cannabis abuse or dependence. They found that 2.3 per 100,000 ED visits in 2006 met criteria compared to 13.3 per 100,000 ED visits in 2013. Men between 20 and 29 years old were the most common demographic, and it was more commonly seen in the west and midwest states compared to the northeast or south.

Multiple treatment options besides providing IV fluids and antiemetics have been tried but are frequently ineffective. These include opioids, benzodiazepines, tricyclic antidepressants, antipsychotics/dopamine antagonists, antiepileptics, antihistamines, corticosteroids, transient receptor potential cation channel subfamily V member 1 agonists (topical capsaicin cream), application of a hot water bottle in the area of abdominal pain, and hot showers and baths, which may not reliably improve the CHS patient’s symptoms [89, 94–96].

One recent retrospective chart review of 247 CHS visits to an academic ED showed that of the ten most frequently administered antiemetics, ondansetron was administered as the first antiemetic 63% of the time but was only effective in 17% of administrations. In contrast, droperidol was effective 48% of the time while haloperidol was at 25%. The most effective pairings were diphenhydramine/metoclopramide (OR = 18) and haloperidol/ondansetron (OR = 10). They did note that 3 patients returned for adverse reactions (2 for haloperidol and 1 for droperidol) [97]. Another study noted that the median length of stay and time to discharge was significantly lower for 37 patients treated with droperidol (most frequently dosed at 0.625 mg IV) compared to 39 without [98]. Taken together, these two studies seem to suggest that the butyrophenones droperidol and haloperidol may be considered as first-line treatment for acute CHS. Supporting this possibility is that both agents are recommended for postoperative nausea and vomiting in consensus guidelines, with haloperidol recommended at 0.5–2 mg IV/IM [99]. Another first-line treatment option might be diphenhydramine/metoclopramide and haloperidol/ondansetron. These recommendations merit further investigation.

One concerning case report documented three patients with CHS who died: a 27-year-old woman, a 27-year-old man, and a 31-year-old man [100]. All had a history of cyclic nausea and vomiting, chronic cannabis use, and negative laboratory and endoscopic findings. All three presented to an ED with nausea and vomiting days before their deaths. CHS was determined to be the cause of death in two of the three patients and contributed to the death in the third patient. For the two who died, both had evidence of renal impairment with elevated kidney function tests.

CHS appears to be increasingly common, appearing the same in other countries [101], and may be missed as a cause of nausea and vomiting [102]. It is important for the clinician to recognize the syndrome (cyclic severe nausea and vomiting for months, associated abdominal pain, negative workups in the past), patient (uses cannabis daily to weekly for > 1 year although it has been reported in acute use as well), male predominance (< 50 years old), and what has worked in the past (improvement with compulsive hot baths, resolution of symptoms when abstaining from cannabis use).

While clinicians may minimize the CHS patient’s symptoms, rare deaths can occur, so we recommend checking electrolytes and administering IV fluids and antiemetics, preferring either of the butyrophenones (droperidol or haloperidol) or combinations diphenhydramine/metoclopramide or haloperidol/ondansetron as initial treatment options, monitoring for adverse reactions with the butyrophenones. The ED physician is reminded that attempts to resolve CHS symptoms may be frequently ineffective and providing education and counseling about cannabis cessation may be the best long-term treatment option.

#### Cannabis withdrawal

##### Clinical scenario

A tremulous 46-year-old woman presents to the ED complaining of fevers and chills, feeling jittery, and unable to sleep since she lost her job last month and can no longer afford to buy medical cannabis that she has been using for her chronic back pain.

Cannabis withdrawal has been described for decades [103–105] but was not recognized in the DSM-5 until its 2013 edition [53]. In a national sub-sample of 1527 regular cannabis users (>3 times a week), a study found a 12% prevalence of withdrawal [106]. The most commonly reported withdrawal symptoms were anxiety/nervousness (76%), hostility (72%), insomnia (68%) and depression (59%). It was associated with mood/anxiety/personality disorders, significant disability and family history of depression. A recent systematic review and meta-analysis reported a 17% prevalence in population-based samples [107]. The criteria for cannabis withdrawal are listed in Table 6. The initial symptoms typically begin within 1–3 days of stopping cannabis, worsen over the first week, and remain for 1–2 weeks in duration, though accompanying insomnia may continue for over a month. Estimates of up to one third of regular cannabis users report withdrawal, which occur in both teenagers and adults but is generally more frequent and severe in the latter [53]. Studies report that women are more likely to suffer from cannabis withdrawal symptoms and suffer worse compared to men [108, 109].

**Table 6:** Criteria for cannabis withdrawal DSM-V^a^

1. Halting of chronic and heavy cannabis use (almost daily to daily use over a minimum of a few months)	
2. Three or more of the following within one week of halting cannabis usage that causes distress or impairment (in social, occupational or other areas of functioning) and is not attributable to another cause: a. irritability, aggression or anger b. anxiety or nervousness c. difficulty sleeping (insomnia or disturbing dreams) d. anorexia or weight loss e. restlessness f. depression g. at least one physical symptom including: fever, chills, headache, abdominal pain, tremors, sweats	

^a^Table adapted from American Psychiatric Association: Diagnostic and Statistical Manual of Mental Disorders: Diagnostic and Statistical Manual of Mental Disorders, Fifth Edition. Arlington, VA: American Psychiatric Association, 2013

While many pharmacotherapies have been tried including antidepressants (selective serotonin reuptake inhibitors and mixed action), anticonvulsants, mood stabilizers, buspirone, *N*-acetylcysteine, and THC preparations, three studies including two review papers [110, 111] and a systematic review [112] found that there was incomplete evidence that any of the pharmacologic treatments was effective for cannabis withdrawal. The latter authors note that THC preparations qualitatively seemed to diminish withdrawal symptoms [113–116], a finding supported by another systematic review on cannabinoid agonist replacement therapy [117]. A final systematic review on the accompanying sleep disturbance associated with cannabis withdrawal that may support abstinence also found insufficient evidence of a pharmacological treatment option [118].

Cannabis withdrawal is therefore considered a diagnosis of exclusion within the appropriate clinical context. Clinicians are advised to initially exclude other emergent conditions associated with symptoms (e.g., fever, chills, headache, abdominal pain) before assessing for an emergent psychiatric state (e.g., suicide, homicide, delusions). Unfortunately, as no current evidence supporting specific treatment options exist, though THC preparations if available may help ease withdrawal symptoms, supportive medical and psychiatric care with education is advised.

#### E-cigarette, or vaping, product use-associated lung injury (EVALI)

##### Clinical scenario

A 19-year-old man presents to the ED complaining of extreme difficulty breathing and nausea for the past few days. When you ask him if he smokes, he answers breathlessly, “no, but I vape.” On further questioning, you learn he buys his products from “friends” and he dabs but has never been to a legal cannabis dispensary. His chest X-ray reveals an infiltrate, but he denies fevers or cough.

In mid-2019, reports began quickly surfacing about a new outbreak of severe pulmonary disease [119]. It was characterized primarily by respiratory symptoms (e.g., shortness of breath, cough, chest pain, observed in 95% of patients) and to a lesser degree gastrointestinal symptoms (such as nausea, vomiting, abdominal pain, and diarrhea that were observed in 77% of patients) that seemed to be related with using electronic cigarettes and vaping products so was termed EVALI [120].

By early 2020, statistics from the CDC showed that most of the EVALI cases were reported in THC containing e-cigarette or vaping products that were obtained from informal sources (such as family members, friends, or dealers) compared to medical or recreational dispensaries [121]. With the finding that vitamin E acetate, a presumed additive in illicit THC containing e-cigarette and vaping products was highly associated to EVALI cases and found in the bronchoalveolar lavage samples of 94% of EVALI patients but not the healthy comparison group, vitamin E acetate was implicated as a likely cause of EVALI [122]. While Vitamin E used topically in skin creams and orally in dietary supplements is not considered harmful, it has not been studied in its aerosolized or inhaled form but based on EVALI is now considered to interrupt normal lung function [123]. Despite the findings related to vitamin E acetate, the CDC has not concluded that other chemicals could be implicated as causal factors as well [123].

The number of cases of EVALI peaked in September 2019 and has been on a steady decline since likely due to public awareness, the removal of vitamin E from products, and law enforcement eliminating illegal products. As of February 18, 2020, there have been 2807 hospitalizations in all US states and 68 deaths reported. Characteristics of afflicted patients include younger (median age 24) males (66%) [123]. Fatalities were higher in non-Hispanic whites, those aged 35 years or older, and those with known histories of asthma, cardiac disease or a mental health condition. Just over 50% of fatalities were in men and in obese individuals [124].

The suggested workup for suspected patients within the appropriate clinical context includes a history of e-cigarette or vaping use, particularly containing THC, and a CXR showing a pulmonary infiltrate without evidence of a pulmonary infection (e.g., negative influenza test, respiratory viral panel, and all other indicated respiratory infectious disease testing) or other plausible alternative diagnoses (e.g., cardiac, neoplastic, or rheumatologic etiology) [125, 126]. Hospital admission, which occurred for 95% of EVALI cases [127], is recommended for patients in respiratory distress or who have O_2_ saturation < 95% on room air. ED treatment should consider the initiation of antibiotic, antiviral, and steroid use empirically along with a cessation of smoking or vaping [120]. While the ED is less likely to experience cases of EVALI now, ED physicians must be aware that the consumption of unused older and particularly illicit products may precipitate EVALI.

#### Synthetic cannabinoids

##### Clinical scenario

A 33-year-old agitated man dressed in army fatigues is brought by police into the ED kicking, screaming, and yelling, “I’m going to kill you jokers!” Police report it took 4 officers to restrain him and that they found a glass pipe in his possession.

Synthetic cannabinoid-receptor agonists (SCRA, aka “synthetic marijuana”) are manufactured cannabinoid receptor agonists that are produced to selectively bind to cannabinoid receptors. Originally conceptualized for medical therapeutic purposes, their use spread to illicit street drugs made to mimic the psychoactive effects of THC without carrying its chemical signature and are often used with the intention to elude detection by urine drug screen assays [128–130]. Some of the initial groups of SCRAs were the HU series (developed at Hebrew University in the 1960s), the CP series (made by Pfizer Inc. in the 1970s), and the JWH series (made by JW Huffman in the 1990s) [128, 129] though the number and variations of SCRAs continue to grow as manufacturers attempt to evade the law [128–131]. The majority of SCRAs possess a higher binding affinity for cannabinoid receptors compared to THC, increasing the risk and duration of psychiatric effects [130] as well as potentially affecting a number of other systems including cardiovascular, renal, gastrointestinal, neurological and neuromuscular, metabolic, and ocular [132].

SCRAs can be sprayed onto dried plant material and smoked like cannabis. In liquid form, it can be vaporized like cannabis. It may be sold on the Internet or in gas stations and convenience stores as herbal products and legal alternatives to cannabis that are touted as safe. They are federally illegal Schedule I drugs, but manufacturers continue to change the chemical formulations to evade the laws. Example names include K2, Spice, Mojo, Cloud 9, Skunk, Joker, Dream, Black Mamba, Crazy Clown, Syn, Haze, and Dragon Spice.

Users of SCRAs may assume the effect will be equivalent to cannabis and provide similar psychoactive effects. They may do so in lieu of cannabis since they believe they can evade a positive drug test for cannabis, which may be a job requirement (e.g., military, athletes, drug treatment program enrollees). And the cost of SCRAs may be a reasonably inexpensive alternative option compared to cannabis.

Because these products are synthesized illegally and comprise a heterogeneous group of chemicals, their effects are unpredictable and may be severe or life-threatening [129, 130, 133–135] The National Poison Data System, CDC and EDs were early reporters of SCRA issues [136–138]. An FDA notice in 2018 warned that synthetic cannabinoids in midwestern states were laced with brodifacoum, an anticoagulant commonly used in rat poison [139], which has not been the only outbreak documented [140, 141]. The risk of needing emergency medical treatment is 14 to 30 times greater for SCRAs than cannabis according to an anonymous online survey of 22,289 drug users in 2012 from 123 countries around the world [142]. One ED found that SCRA overdoses had significantly more cardiotoxic and neurotoxic effects compared with cannabis [143].

A systematic review of scientific reports on the adverse events of SCRAs identified about 4000 cases of SCRA exposures [144]. The typical patient is a young male (59–100%) who presents with tachycardia (37–77%), agitation (16–41%), and nausea (13–94%). Most cases were observed in an acute setting, received supportive care (e.g., oxygen, IV fluids, benzodiazepines) and discharged within 8 h. However, there were an estimated 22–27 deaths due to SCRAs. Major complications noted in the study were cardiovascular (e.g., MI, stroke), acute kidney injury, tonic-clonic seizures, nausea/vomiting, and psychiatric problems (e.g., agitation, psychosis, panic attack, anxiety, paranoia, hallucinations, suicidality).

In early 2015, the CDC was notified about increasing calls to the US poison centers regarding synthetic cannabinoid use [145]. Monthly calls from January 2015 to April 2015 climbed from 349 to 1501, a 330% increase. A total of 3572 calls were received in the first 5 months of 2015. Similar to the systematic review above, calls were typically about males (80.7%) with a median age 26. The most common adverse effects reported were agitation (35.3%), tachycardia (29.0%), drowsiness or lethargy (26.3%), vomiting (16.4%), and confusion (4.2%).

The majority reported smoking (80.3%) compared to ingesting (19.5%). Most usage was intentional (92.7%). Co-ingestants were reported in 17.5% of cases. Of those, alcohol (23.0%), plant-derived cannabis (16.5%), and benzodiazepines (11.0%) were most common. Major adverse effects, defined as potentially life-threatening or leading to permanent disability, were seen in 11.3%; moderate effects, defined as requiring some form of treatment, were seen in 47.5%; minor effects, defined as minimal results that were rapidly resolving, were seen in 37.0%; and 3.7% had no effect. Fifteen deaths (0.5%) were reported. Callers aged 30+ were significantly more likely than those aged 10–19 to have a severe outcome.

In summary, the clinician should not confuse a cannabis exposure with a synthetic cannabinoid exposure, the latter of which is more likely to be severe and life-threatening. There may be overlapping signs and symptoms and no clear way to distinguish between the two other than a detailed history. Questions regarding urine drug testing for employment purposes may be helpful in sorting out particular use and motivation. Exposed patients may be buying on the rumor (fake is safe and undetectable) but may abstain with education (fake is not safe and increasingly likely to be detected). The clinician should assess for co-ingestants that may impact care. A standard urine drug screen will not detect synthetic cannabinoids, but there are reports of laboratories working actively on developing tests for multiple synthetic metabolites [146–149]. Clinicians who verify a SCRA exposure need to be vigilant about providing supportive care, paying particular attention to cardiovascular, renal, gastrointestinal, neurologic, and psychiatric manifestations that may necessitate checking laboratory studies including electrolytes (particularly blood urea nitrogen and creatinine), creatine kinase, troponin, complete blood count, alcohol level, aspirin level, acetaminophen level, coagulation tests, and a urine drug screen test.

## Conclusion

Cannabis itself is generally considered low risk compared to tobacco, alcohol, and other illicit drugs [150]. However, with increasing availability due to state legalization, increasing percentages of THC in botanicals and newly developed cannabis concentrates, this may be changing. Because of the new higher concentrated products, which can be in the hundreds to low thousands of milligrams of THC, our understanding of the effects due to ingestion and inhalation is still not well studied or understood.

Regulated state markets should oversee commerce to ensure public safety and welfare. This can be achieved through interstate (or eventually federal if cannabis were to become legal nationwide) laws that require only safe cannabis enters the marketplace (e.g., without pesticides, microbial impurities, contaminants, adulterants). Product safeguards could include limiting the total quantity of cannabis per product and serving size, limiting the amount a single consumer can purchase, overseeing package quantities are clearly labeled and accurate, and regulating advertising, product design, and child-resistant packaging. Many of these changes have already occurred in California and Colorado. Furthermore, states may consider limiting the taxes that they impose on cannabis that drives up the legal market prices by as much as 47% [151] and rewards the sales and distribution of cheaper illegal products such as synthetic cannabinoids and products that caused EVALI.

We would hope that individual physicians, professional medical societies, and medical specialties would work to educate patients about the risks and benefits of using medical cannabis, which necessitates advocating for further research to clarify the effects of this ubiquitous product. As it stands, most physicians have limited training [152] or practical knowledge of medical cannabis [153] or cannabis laws [154]. We believe that cannabis dispensary personnel should receive mandatory education from physicians that teach uniform standards for safe use. Concurrently, we would like consumers to be referred to trained medical professionals for cannabis education and discourage the practice of allowing medical advice from untrained dispensary personnel.

Unfortunately, due to the requirements placed by new state regulations driving up the price in the regulated market, there will always be a cheaper black market for both cannabis (particularly that which has bypassed state regulations requiring seed to sale tracking—e.g., remember EVALI) and synthetic cannabinoids; and thus, ED physicians must be aware of what exists even beyond the capture of the regulated markets.

We recommend the following approach for the ED patient who presents with potential cannabis-related issues. First, stabilize the patient and assess the need for airway protection or intubation, particularly in infants and toddlers. If the patient has respiratory distress or their O_2_ saturation is < 95%, consider EVALI. Likewise, if the patient has a severe psychiatric reaction, consider synthetic cannabinoids. Take a careful history of use to distinguish cannabis versus synthetic cannabinoid usage and regulated versus unregulated sources of product. Second, determine the product type, amount of THC consumed, mode of consumption, and assess for other co-ingestants. Maintain a high suspicion for unintentional pediatric patient exposure for undifferentiated AMS, ataxic, or sleepy patients who were previously healthy, paying particular attention to the type and amount of THC and mode consumed per kilogram of body weight. Third, understand CHS and cannabis withdrawal, recalling that CHS can occasionally be fatal. Fourth, consider EVALI in patients with any respiratory or gastrointestinal complaints in the proper clinical context. Lastly, do not let the bias of cannabis usage impede the consideration of cardiac etiologies, AMS diagnostic potentials, intentional overdoses, rare but potentially life-threatening cases of CHS, EVALI, and synthetic cannabinoid presentations.

## Article summary

1. Why is the topic important? Cannabis is being used increasingly for both medical and adult/recreational purposes. Newer more potent strains, concentrated THC products, and consumption methods have changed the ED management of cannabis, and physicians must be able to identify the truly emergent cannabis intoxicated patient.

2. What does this clinical review attempt to show? We categorize six areas for EM physicians to consider cannabis ingestion and provide descriptions and a treatment approach to each of the areas.

3. What are the key findings? Physicians must determine the type and mode of cannabis ingestion to assess how it differentially affects adults compared to pediatric patients. Long-term cannabis use may cause patients to seek ED care. Treating patients who have used unregulated or synthetic cannabinoids may be very different from those who have used cannabis.

4. How is patient care impacted? The ED physicians must be able to discern not only the effects of cannabis usage on pediatric and adult patients but consider other factors such as co-ingestions and intentional ingestions as well as understand treatment strategies.

## Data Availability

Not applicable
